# Syncope Secondary to Concomitant Ingestion of Tizanidine and Alcohol in a Patient With Alcohol Use Disorder

**DOI:** 10.7759/cureus.57249

**Published:** 2024-03-30

**Authors:** Sabastain F Forsah, Derek Ugwendum, Divine Besong Arrey Agbor, Nancelle Ndema, Nkafu Bechem Ndemazie, Gauvain Kankeu Tonpouwo, Akua Aboah A Taylor, Nkeng Fuoching, Davene James-Gregory, Shannia Amoah, Vaithilingam Arulthasan, Jay Nfonoyim

**Affiliations:** 1 Internal Medicine, Richmond University Medical Center, Staten Island, USA; 2 Pulmonary and Critical Care, Richmond University Medical Center, Staten Island, USA

**Keywords:** central nervous system, alcohol use disorder, tizanidine, alcohol, orthostatic hypotension, syncope

## Abstract

Syncope is the transient loss of consciousness due to cerebral hypoperfusion. A significant number of individuals experience a syncopal attack at one stage of their lives. The common causes of syncope include vasovagal syncope, orthostatic hypotension, and cardiac causes. Drugs are also associated with causing syncope. The drugs involved are mostly those that depress the central nervous system, and concomitant use of more than one of such drugs increases the risk of syncope even further. Tizanidine and alcohol individually can cause hypotension and combining both drugs is not advised due to heightened central nervous system depression and profound hypotension. We present a case of a 53-year-old female with alcohol use disorder who presented with a first-time syncopal attack due to postural hypotension after ingesting both tizanidine and alcohol concurrently. Co-administration of tizanidine and alcohol is not advised, however, cases of syncope have been rarely reported with concomitant use. This case will enlighten physicians to counsel patients about the need to abstain from alcohol consumption when taking tizanidine.

## Introduction

Syncope is the transient loss of postural tone and consciousness due to global cerebral hypoperfusion. It is characterized by sudden onset followed by rapid and usually complete recovery [1]. About 41% of individuals experience at least one syncopal episode during their lifetime [2,3]. Its prevalence increases with age and it is an important cause of morbidity and mortality especially in the older population [4]. Syncope is responsible for about 3% of all emergency room visits and 6% of all hospitalizations. Among the causes of syncope, vasovagal syncope, orthostatic hypotension, and cardiac syncope are the most common [3]. Drugs have also been implicated as causes of syncope and include any medication with the potential to cause depression of the central nervous system. A combination of two or more such drugs can increase the risk of a syncopal attack even further [4]. We present a case of a 53-year-old female who was brought to the emergency room (ER) for a syncopal episode after ingesting both alcohol and a therapeutic dose of tizanidine. This case is unique because, although alcohol consumption is not advised when taking tizanidine due to postural hypotension [5], cases of syncope after coadministration of tizanidine and alcohol have rarely been reported. This case presentation will enable physicians to emphasize to patients the necessity to avoid alcohol consumption when taking tizanidine.

## Case presentation

A 53-year-old female was brought to the emergency room after a first-time syncopal episode. She does not know for how long she was down but her daughter confirmed that she fully gained consciousness in the ambulance after a period of some confusion. No seizures were noted. The morning before the syncopal attack, she took her newly prescribed medication for sciatica together with alcohol. Some hours later, while at the grocery store, she started feeling lightheaded and fainted. The patient has a past medical history of asthma, sciatica, and alcohol use disorder (she is an everyday drinker), with many admissions for alcohol withdrawal syndrome. She works as a bartender and recently started tizanidine 4 mg daily for sciatica. The patient was on no other medication. Initial vital signs were blood pressure (BP) 169/84 mmHg, heart rate 86/minute, respiratory rate 16/minute, and body mass index 23 Kg/m2. Further evaluation revealed that the patient’s orthostatic vital signs were positive (BP lying was 157/89, BP sitting was 141/93, and BP standing was 132/78). Physical examination on arrival was unremarkable. The significant laboratory investigations are shown in Table 1. The laboratory values were indicative of chronic alcoholism with acute intoxication. Additionally, the urine drug screen was negative. The folic acid level was obtained after the patient was given a banana bag and folic acid.

**Table 1:** Significant initial laboratory results ALT, alanine aminotransferase; AST, aspartate aminotransferase

Laboratory investigation	Reference value	Patient’s result
Hematology		
White cell count (K/UL)	4 – 11.2	6.1
Hemoglobin (g/dL)	11.2 – 15.7	10.9
Mean cell volume (fl)	79 – 98	101.9
Platelets (K/UL)	150 - 400	138
Chemistry		
Sodium (mmol/L)	136 – 145	125
Potassium (mmol/L)	3.5 – 5.1	3.2
Magnesium (mg/dL)	1.6 – 2.6	1.16
Blood glucose (mg/dL)	74 – 106	126
Albumin (g/dL)	3.2 – 4.8	4
AST (U/L)	< 34	82
ALT (U/L)	10 – 49	36
Total bilirubin (mg/dL)	0.2 – 1.0	0.9
Direct bilirubin (mg/dL)	0.0 – 0.3	0.4
Creatine kinase (U/L)	34 – 145	169
Lactic acid (mg/dL)	0.5-2.2	3.1
Folic acid (ng/mL)	> 5.38	>24
Vitamin B12 (pg/mL)	211 – 911	715
Toxicology		
Serum ethyl alcohol (mg/dL)	0	172

**Figure 1:**
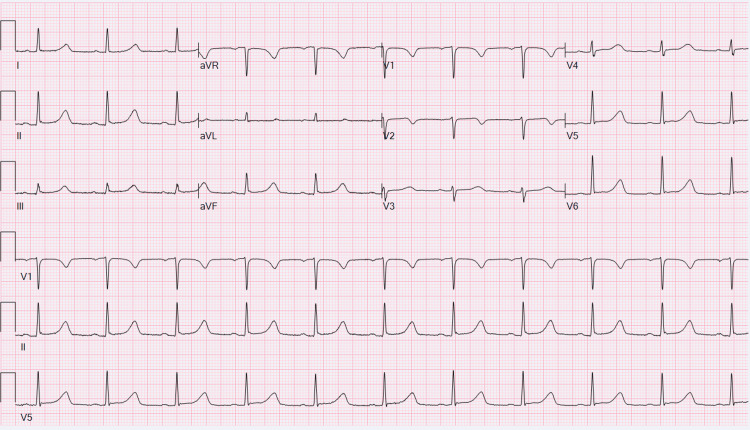
EKG showing normal sinus rhythm with prolonged QTc interval

Electrocardiogram (EKG) showed normal sinus rhythm with a prolonged QTc interval of 528 ms (Figure 1). QTc interval progressively got shorter during hospitalization. Telemetry monitoring did not show any arrhythmia. Echocardiography was normal.

**Figure 2:**
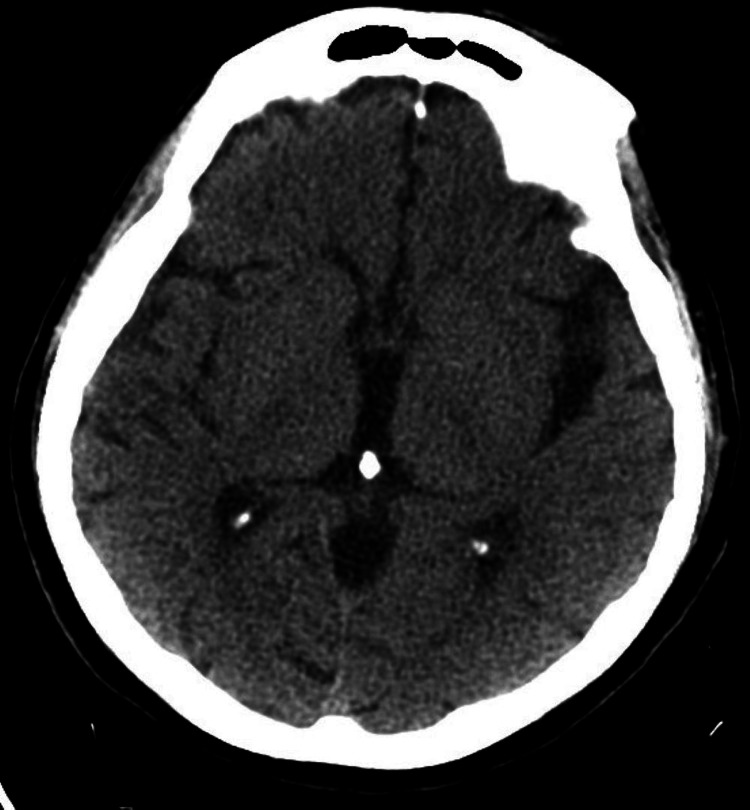
CT brain showing no evidence of acute hemorrhage or infarct, no midline shift, no mass effect, no extra-axial fluid collection and normal osseous structures

Computed tomography (CT) of the brain showed no acute abnormality (Figure 2) and electroencephalogram (EEG) was negative for seizures.

The patient was initially admitted for observation. However, the hospital course was marked by severe alcohol withdrawal syndrome needing ICU care, and after improvement, she was downgraded to the general medical floor. She was treated with chlordiazepoxide, intravenous fluids, electrolytes, and vitamins, including thiamine and folic acid. Alcohol withdrawal symptoms resolved, but the patient persistently had dizziness on waking up from seated to standing position with a tilt table test positive for orthostatic hypotension. The patient was started on metoprolol and fludrocortisone and the postural hypotension resolved. She was counselled on alcohol cessation and was provided with a referral to outpatient alcohol cessation support groups upon discharge.

## Discussion

Globally, alcohol is the most commonly used recreational drug and the most abused drug among the adult population. It accounts for more than 3.3 million deaths per year worldwide [6]. In 2018, 14.4 million people suffered from alcohol use disorder (AUD) in the US, with alcohol accounting for about 100,000 deaths [7]. Alcohol can easily cross membrane barriers including the blood-brain barrier, and reach different parts of the body. Excessive alcohol consumption is responsible for causing more than 200 disorders including gastritis, pancreatitis, cardiomyopathies, hypoglycemia, alcoholic hepatitis, macrocytic anemia, liver cirrhosis, cancer, electrolyte imbalances and neurocognitive impairment [6,8]. One of the most common consequences of alcohol consumption, especially when consumed in excess, is its ability to depress the central nervous system (CNS). It depresses the CNS by enhancing the effect of the inhibitory neurotransmitter, gamma-aminobutyric acid (GABA). It also inhibits the actions of glutamate on the N-methyl-D-aspartate (NMDA) receptors, which can cause slurred speech, stupor, and gait abnormalities and may even result in a coma [8]. Alcohol can also cause hypotension [9]. The mechanism by which alcohol causes syncope is not well known. However, a possible explanation is that alcohol intake causes orthostatic hypotension because of an impairment in the vasoconstrictor response to orthostatic stress which can then lead to syncope [10]. Our patient had been drinking for a long time but had never experienced a syncopal episode, and only did when she ingested tizanidine concurrently with alcohol. 

Tizanidine is a centrally acting alpha 2 receptor agonist. It also inhibits the release of excitatory amino acids like glutamate and aspartate from spinal interneurons with the resultant effect being the enhancement of the presynaptic inhibition of motor neurons. It is a centrally acting skeletal muscle relaxant and is used for the symptomatic treatment of painful muscle spasms and spasticity and can also be used for headaches [5,11]. The oral bioavailability of tizanidine is about 21% mainly due to extensive first-pass metabolism by the cytochrome (CYP) P450 - 1A2 enzyme in the liver while alcohol is metabolized by the CYP2E1 [12]. Using tizanidine with other CYP1A2 inhibitors such as oral contraceptives, dronedarone, pimozide, saquinavir, cimetidine, famotidine, and acyclovir should be avoided due to decreased clearance of tizanidine with risk of increased toxicity [13].

Tizanidine's side effects include lethargy, bradycardia, hypotension, drowsiness, agitation, confusion, and even coma. Most of the side effects of tizanidine manifest at toxic doses, and therapeutic doses are more tolerated. However, when combined with other medications even at therapeutic doses, side effects become more evident like in our patient [14]. Tizanidine can interact with other medications like fluvoxamine, ciprofloxacin, and benzodiazepines and cause more profound hypotension [15]. likewise, Like in our patient, tizanidine when taken with alcohol can potentiate the side effects of alcohol leading to postural hypotension, excessive sedation and myocardial toxicity, like bradycardia [5,16]. Our patient had a first-time syncopal episode due to profound postural hypotension secondary to the synergistic effect of tizanidine and alcohol on lowering blood pressure.

Syncope can be diagnosed with various modalities. Neuronal-mediated syncope can be diagnosed with carotid sinus massage and autonomic nerve function test. Postural hypotension can be diagnosed with a tilt table test and with postural vital signs. Cardiac causes of syncope can be diagnosed with EKG monitoring, video recording of suspected syncope, electrophysiological examination, endogenous adenosine assessment, echocardiography, exercise stress test and coronary angiography. An EEG and brain imaging can be used to rule out mimickers of syncope-like seizures and cerebrovascular accidents respectively [2]. However, a specific diagnosis is not obtained in about half of patients presenting with syncope [17].

Treatment of syncope is etiology-specific. Syncope resulting from alcohol and tizanidine concomitant ingestion involves supportive care, including fluid resuscitation, alcohol cessation, and reduction in the dose of tizanidine. It is very important to educate patients about avoiding alcohol ingestion when taking tizanidine [5,9].

## Conclusions

Drinking alcohol while on tizanidine should be strongly discouraged and patients should be counselled about the side effects of ingesting both tizanidine and alcohol together. Tizanidine and alcohol ingestion can cause significant postural hypotension which can lead to syncope. Syncope can cause significant morbidity and mortality, especially in the elderly, and it is a fairly common cause of emergency visits and hospitalization. Reducing the causes of syncope can go a long way to decreasing its burden.
